# PAI-1 protein is a key molecular effector in the transition from normal to PTSD-like fear memory

**DOI:** 10.1038/s41380-021-01024-1

**Published:** 2021-01-28

**Authors:** C. Bouarab, V. Roullot-Lacarrière, M. Vallée, A. Le Roux, C. Guette, M. Mennesson, A. Marighetto, A. Desmedt, P. V. Piazza, J. M. Revest

**Affiliations:** 1 Univ. Bordeaux, INSERM, Neurocentre Magendie, U1215, F-33000 Bordeaux, France; 2 Present Address: Aelis Farma, 33077 Bordeaux, France

**Keywords:** Neuroscience, Psychiatric disorders

## Abstract

Moderate stress increases memory and facilitates adaptation. In contrast, intense stress can induce pathological memories as observed in post-traumatic stress disorders (PTSD). A shift in the balance between the expression of tPA and PAI-1 proteins is responsible for this transition. In conditions of moderate stress, glucocorticoid hormones increase the expression of the tPA protein in the hippocampal brain region which by triggering the Erk1/2^MAPK^ signaling cascade strengthens memory. When stress is particularly intense, very high levels of glucocorticoid hormones then increase the production of PAI-1 protein, which by blocking the activity of tPA induces PTSD-like memories. PAI-1 levels after trauma could be a predictive biomarker of the subsequent appearance of PTSD and pharmacological inhibition of PAI-1 activity a new therapeutic approach to this debilitating condition.

## Introduction

Stressful events trigger a set of biological responses which generally increase adaptation to potentially harmful situations. However, overly intense or chronic stress can have deleterious effects leading to several behavioral disorders including substance use disorders, depressive-like and anxiety-like disorders, in particular post-traumatic stress disorder (PTSD) [1–4]. Memory performances are a prototypical example of this dichotomy between the beneficial and pathological effects of stress [5]. Moderate stress increases the memory of associated events facilitating adaptation to future similar situations [2, 3, 6]. In contrast, intense stress can alter memory consolidation leading to pathological conditions such as PTSD [3, 4, 7, 8]. PTSD is a severe stress-related disorder with an estimated lifetime prevalence of about 8% in the general population. Thirty percent of victims of traumatic events (e.g., rape, terrorist attacks, military combat, genocide) develop this condition with this figure rising to 50% in the most severe situations. Prevalence is also dependent on biological sex differences [9–13]. PTSD is characterized by recurrent and intrusive recollections of the trauma (i.e., emotional hypermnesia) due to the inability of the individual to restrict fear to the appropriate predictor of the threat [7, 9, 13]. Amygdalar hyperactivation was shown to contribute to PTSD-related emotional hypermnesia [9, 14–17]. However, PTSD was also shown to be associated with a hippocampal dysfunction that might contribute to the deficit of contextual memory of the trauma, which in turn would cause the formation and persistence of PTSD-related hypermnesia [18*–*21]. This progressive shift from adaptive to deleterious consequences as a function of stress intensity follows an inverted-U pattern and has been known since the beginning of the twentieth century [22*–*24], but the molecular mechanisms of this pathophysiological process, and notably those underlying the shift from adaptive to maladaptive (PTSD-like) fear memory, remain largely unknown.

It has been suggested that glucocorticoid hormones (GC), one of the major biological responses to stress, may be one of the factors involved in the shift from beneficial to pathological effects of stress [25]. The increase in GC induced by moderate stressors enhances the memory of stress-associated events [26*–*31] whilst a dysregulation of the hypothalamic-pituitary-adrenal (HPA) axis and functional alterations of the glucocorticoid receptor (GR) have been consistently related to PTSD-like memory impairments [7, 32, 33]. Both effects of GC are mediated by the activation in the brain of the GR, a hormone-activated transcription factor belonging to the family of nuclear receptors [34]. We have previously shown that in conditions of moderate stress, the activation of the GR in the hippocampus, one of the major brain structures involved in memory processing and in PTSD pathophysiology [19, 21, 35, 36], induces a cascade of molecular events referred to as the GMES signaling cascade that increases memory formation. The first step is the increase of tPA (tissue plasminogen activator)-activated plasmin which cleaves the pro-BDNF to mature BDNF. Mature BDNF, by activating the TrkB receptor, phosphorylates Erk1/2^MAPK^ which, increasing the expression of the downstream transcription factor Egr-1, finally enhances the levels of memory-enhancing effector proteins, such as synapsin Ia/Ib [26*–*28]. In this report, combining complementary in vitro/in vivo molecular and pharmacological approaches and the first mouse model recapitulating the two memory components of PTSD (i.e., emotional hypermnesia and contextual amnesia) [5], we show that a deregulation of the GMES signaling cascade in the dorsal hippocampus, triggered by an increase in GC-induced type 1 plasminogen activator inhibitor (PAI-1) levels, underlies the transition from a normal memory to a PTSD-like fear memory.

## Materials and methods

### Chemicals

In all the experiments, we used a preformed water-soluble complex of corticosterone (Cort) and 2-hydroxypropyl-β-cyclodextrin (#C174, Sigma, USA) [26*–*28]. In mice, Cort (2 mg/kg in a volume of 0.1 ml/10 g body weight) or vehicle (NaCl 0.9%) was administered i.p. immediately after the acquisition of fear conditioning in order to mimic the effect of intense trauma [7]. Cort was used at 100 and 1000 nM on rat PC12 cells and at 10 and 1000 nM on rat hippocampal slices [26*–*28]. Millipore (USA) provided the recombinant human BDNF (CAS Nb 218441-99-7, #GF029, 100 ng/side) and tPA inhibitor, stable recombinant mutant of human PAI-1 (CAS Nb: 140208-23-7, #528208, ranging from 30 to 240 ng/side) [28, 37]. The small-molecule inhibitor of PAI-1 activity tiplaxtinin (PAI-039; CAS Nb: 393105-53-8, #1383, 5 ng/side) was provided by Axon MEDCHEM (The Netherlands).

### Cell culture

**Fig. 1:**
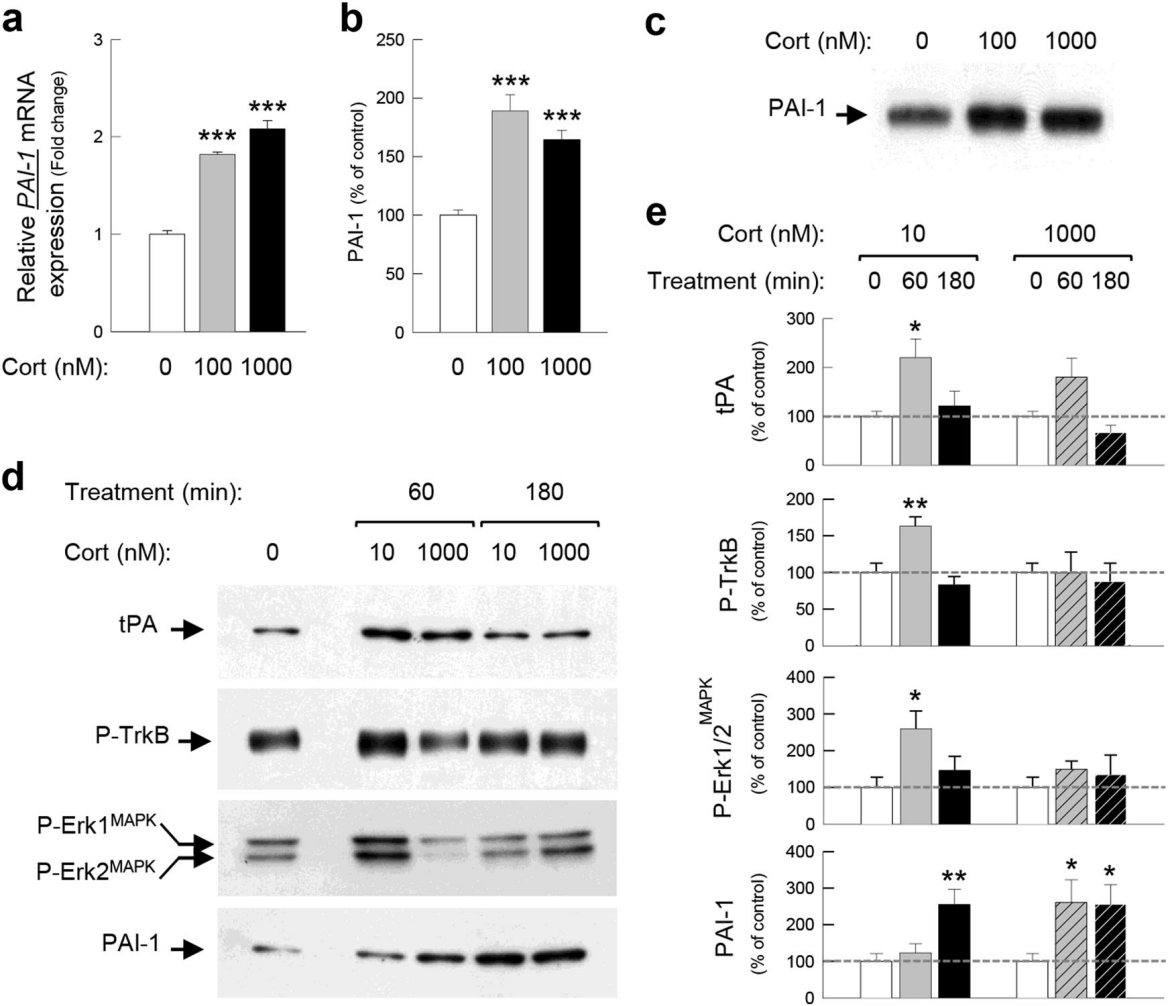
PAI-1 expression is increased by corticosterone. PAI-1 mRNA, measured by qPCR (**a**), and protein expressions, measured by western blot (**b**, **c**), in response to 100 and 1000 nM of Cort for 3 h (180 min) in PC12 cells. PAI-1, tPA, P-TrkB, and P-Erk1/2^MAPK^ proteins measured by western blot (**d**, **e**) in dorsal hippocampal slices of Sprague-Dawley rats incubated with 10 and 1000 nM of Cort for 1 h (60 min) and 3 h (180 min). α-tubulin and βIII-tubulin were used as a loading control. X-ray films were quantified by densitometry (OD). Dunnett’s multiple comparisons post hoc test after ANOVA: *p < 0.05, **p < 0.01, ***p < 0.005 compared to control conditions. Plotted values are means ± sem.

PC12 cell line (ATCC CRL-1721) derived from a transplantable rat pheochromocytoma was used [26, 28]. PC12 cells were seeded on six-well plates coated with poly-D-lysine at the appropriate concentration (10^5^ cells/well) in fresh, antibiotic-free medium (DMEM/F12 (#31330-038, Gibco, USA) + 10% fetal bovine serum (#10270106, Fisher Scientific, USA)). Sixteen hours before Cort treatment, the medium was changed for a steroid-free culture medium (DMEM/F12 + 10% charcoal/dextran-treated FBS (#SH30068-03, Hyclone, Fisher Scientific, USA). PC12 cells (*n* = 5–6/group) were treated with 100 and 1000 nM of Cort-HBC (Sigma, USA), then harvested after 3 h and the proteins and RNA extracted (Fig. 1 and Fig. S1).

### Hippocampal slice preparations and Cort treatment

Hippocampal slice preparations have been described in detail previously [38]. Briefly, adult male Sprague-Dawley rats (2–3 months old, *n* = 18, Charles River Laboratory, France) were used. Rats were anesthetized with isoflurane and transcardially perfused with nearly frozen modified artificial cerebrospinal fluid (CSF) with 3 mM kynurenic acid. The modified CSF for perfusion contained: (in mM) 87 NaCl, 75 sucrose, 25 glucose, 5 KCl, 21 MgCl_2_, 0.5 CaCl_2_, and 1.25 NaH_2_PO_4_. After perfusion, the brains were quickly removed and sliced (300 μm) in the coronal plane using a vibratome (Campden Instruments, UK). Immediately after cutting, slices were stored for 40 min at 32 °C in CSF ((in mM): 130 NaCl, 11 glucose, 2.5 KCl, 2.4 MgCl_2_, 1.2 CaCl_2_, 23 NaHCO_3_, 1.2 NaH_2_PO_4_), equilibrated with 95% O_2_/5% CO_2_ then stored at room temperature for the rest of the experiment. Each brain slice was then treated for 1 h (60 min) and 3 h (180 min) with 10 and 1000 nM of Cort. To address the molecular mechanism of the bell-shaped effects of Cort, we used 10 nM of Cort shown to promote the GMES signaling cascade [26*–*28] and 1000 nM of Cort to reveal the harmful effects of Cort, corresponding to the descending limb of the bell-shaped dose response function of this hormone [39, 40]. One slice served as a control reference and did not undergo any treatment. Dorsal hippocampi were isolated, and proteins were extracted as previously described [26, 28] (Fig. 1 and Fig. S1).

### Protein extraction from brain tissues and immunoblotting analysis

A detailed description of protein extraction and immunoblotting analysis has been reported previously [26*–*28, 41, 42]. Briefly, protein sample extracts from PC12 cells and mouse and rat hippocampi were performed in RIPA buffer containing protease and phosphatase inhibitors (#P8340 and #P0044, Sigma, USA) before being subjected to immunoblotting experiments. SDS-PAGE-separated proteins were then revealed with relevant antibodies. Rabbit polyclonal anti-PAI-1 antibodies were from Lifespan Biosciences (LSBio#C81062, 1/1000, WA, USA) and Epitomics (#3917-1, 1/3000, CA, USA), anti-tPA (#T5600-05G; 1/5000) was from US Biological (MA, USA), anti-Erk1/2^MAPK^ (#06-182; 1/50000) was from Millipore (MA, USA), and anti-Phospho-Erk1/2^MAPK^ (#9101S; 1/1000) was from CST (MA, USA). Rabbit monoclonal antibodies anti-Phospho-Erk1/2^MAPK^ (#4370; 1/5000) was from CST (MA, USA), anti-phospho-TrkB (#2149-1; 1/5000) was from Epitomics (CA, USA) and anti-TrkB (#610101; 1/2000) was from BD Biosciences (NJ, USA). Mouse monoclonal anti-neuronal class III β-tubulin (TUJ1) (#MMS-435P; 1/20000) was from Eurogentec (Belgium) and anti-α-tubulin (#N356, 1/50000) was from Amersham Life Sciences (Del, USA). CST provided secondary antibodies: anti-rabbit IgG, HRP-linked antibody (#7074, 1/5000), and anti-mouse IgG, HRP-linked antibody (#7076, 1/20000). In all experiments, βIII-tubulin or α-tubulin measures were used as a loading control. X-ray films (Kodak, USA) were quantified by densitometry (optical density; OD) using a GS-800 scanner coupled with Quantity One software (Bio-Rad, CA, USA).

### Quantitative PCR analysis

Samples of PC12 cells treated with Cort were homogenized in Tri Reagent (Euromedex, France) and RNA was isolated using a standard chloroform/isopropanol protocol [43]. RNA was processed and analyzed using an adapted version of published methods [44]. cDNA was synthesized from 2 μg of total RNA using RevertAid Premium Reverse Transcriptase (Fermentas, Thermo Fisher Scientific, USA) and primed with oligo-dT primers (Fermentas, Thermo Fisher Scientific, USA) and random primers (Fermentas, Thermo Fisher Scientific, USA). qPCR was performed using a LightCycler^®^ 480 Real-Time PCR System (Roche, Meylan, France). qPCR reactions were done in duplicate for each sample, using transcript-specific primers, cDNA (4 ng), and LightCycler 480 SYBR Green I Master (Roche) in a final volume of 10 μl. The PCR data were exported and analyzed using a computer-based tool (Gene Expression Analysis Software Environment) developed at the Neurocentre Magendie (France). The GeNorm method was used to determine the reference gene. Relative expression analysis was corrected for PCR efficiency and normalized against two reference genes. The ribosomal protein L13a (*Rpl13a*) and non-POU-domain-containing (*Nono*) genes were used as reference genes. The relative level of expression was calculated using the comparative (2^−∆∆CT^) method [45]. qPCR amplification used specific primers to specifically amplify *Serpine1* gene encoding PAI-1 protein and *Nono* and *Rpl13a* as reference genes (Fig. 1).

PAI-1 forward primer sequence (5′-3′): GGCACAATCCAACAGAGACAATC and reverse primer sequence (5′-3′): AGGCTTCTCATCCCACTCTCAA.

Nono forward primer sequence (5′-3′): AACAGGGTTGCTGTGTGTTGAA and reverse primer sequence (5′-3′): TGCACAGCGCAACTACCTAAGATA.

Rpl13a forward primer sequence (5′-3′): TGGAGAAGAAAATCTGCAAGTTCA and reverse primer sequence (5′-3′): TCTTTATTGGGTTCACACCAAGAGT.

### Restraint stress

**Fig. 2:**
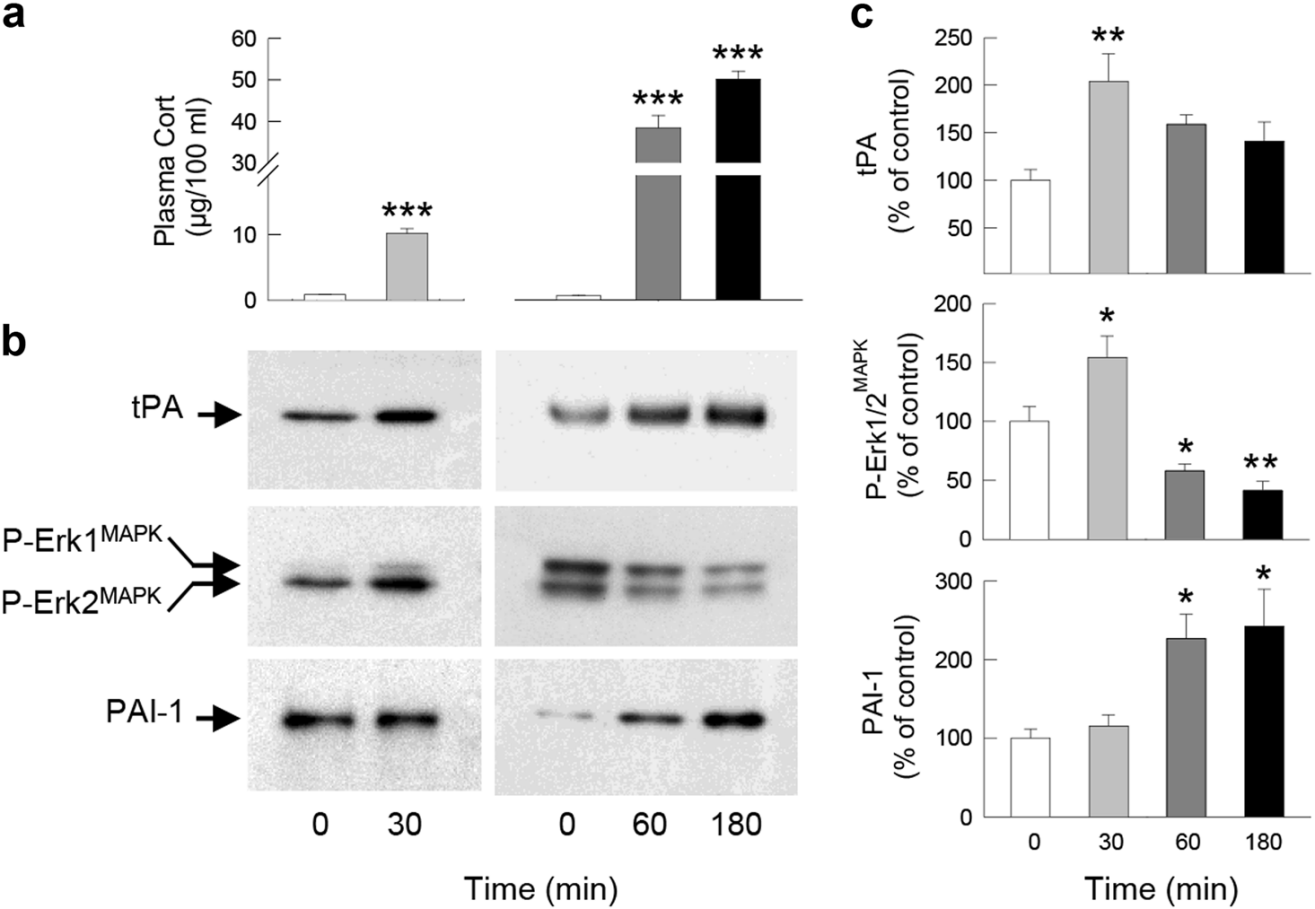
PAI-1 expression is increased by stress. Plasma corticosterone levels (**a**), PAI-1, tPA, and P-Erk1/2^MAPK^ proteins measured by western blot (**b**, **c**) in the dorsal hippocampus of C57BL/6J mice in response to 30 min, 1 h (60 min), and 3 h (180 min) restraint stress. βIII-tubulin was used as a loading control. X-ray films were quantified by densitometry (OD). Dunnett’s multiple comparisons post hoc test after ANOVA: *p < 0.05, **p < 0.01, ***p < 0.005 compared to control conditions. Plotted values are means ± sem.

Male C57BL/6J mice aged 2–3 months old (*n* = 30) (Fig. 2) were obtained from Charles River Laboratory, France. Mice were placed into 50 ml conical centrifuge tubes fitted with a central puncture so as to allow ventilation. The tubes were placed in horizontal holders with strong light exposure, and the animals were held in this way for a continuous period of restraint. After 30 min, 1 h (60 min), and 3 h (180 min) of restraint, the mice and those from an unstressed control group were sacrificed by decapitation, then the hippocampi and blood were collected and assayed for protein extraction [28, 42].

### Blood collection for Cort assay

Blood was rapidly collected in heparin-EDTA-coated tubes (Sarstedt, France) and centrifuged at 2000 rpm (4 °C, 20 min). Supernatant containing the blood plasma was stored at −20 °C, and then processed for Cort assay. Plasma Cort (Corticosterone EIA kit #KO14-H1, Arbor Assays, Michigan, USA) levels were quantified by ELISA following the manufacturer’s instructions [28, 42].

### Behavioral procedure

#### Surgical procedure

**Fig. 3:**
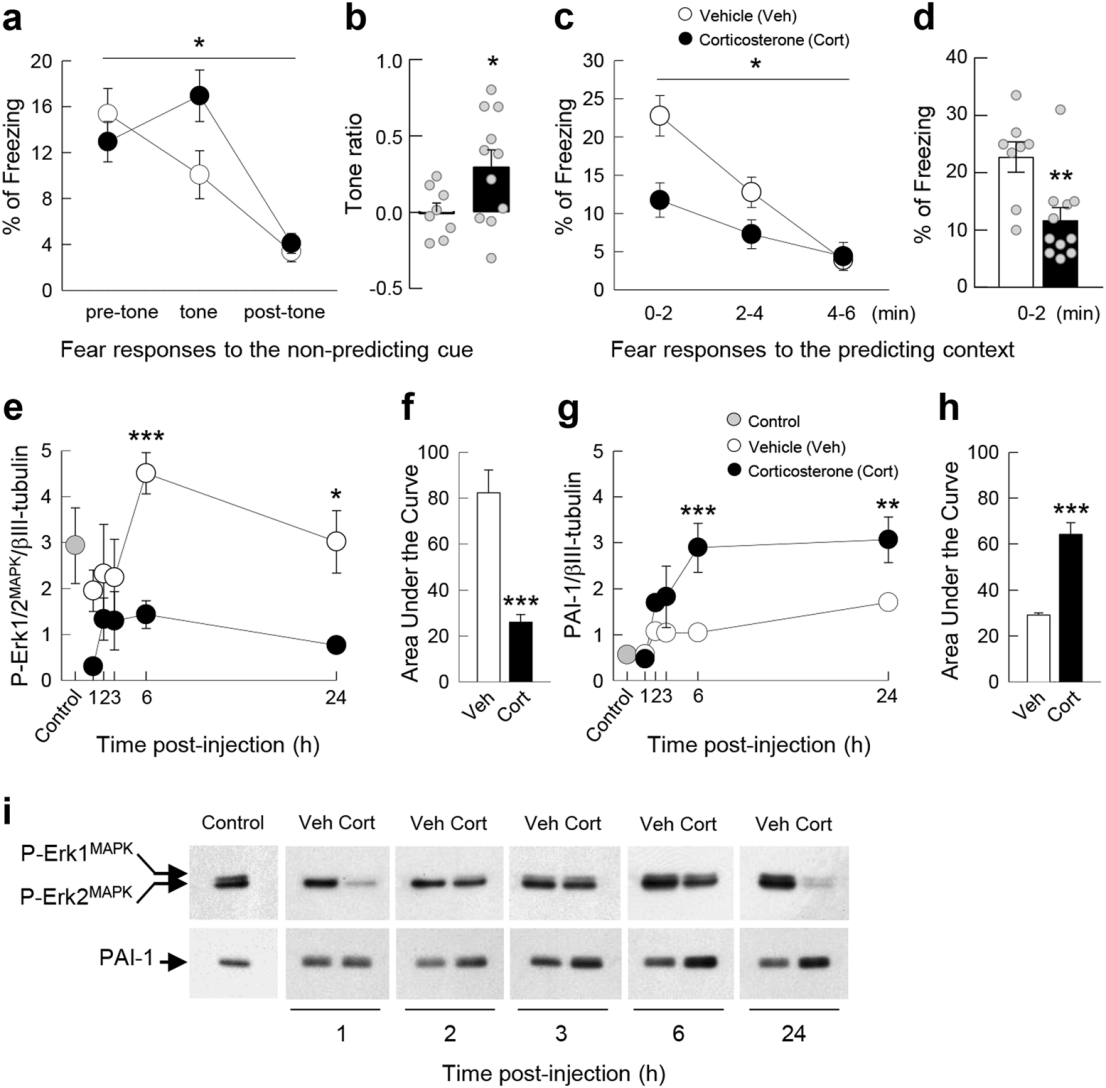
The development of PTSD-like memory is associated with an increase in PAI-1 expression. Fear responses, expressed as percent of time spent freezing, 24 h after conditioning in C57BL/6J mice exposed in a safe environment to the tone not predicting the threat (nonpredicting cue, **a** and **b**) or to the environment in which the conditioning was performed (predicting context, **c** and **d**). Expression of P-Erk1/2^MAPK^ (**e**, **f**) and PAI-1 (**g**, **h**) proteins in the dorsal hippocampus at different times after the conditioning sessions (**e**, **g**) and expressed as area under the curve encompassing the 24 h of analysis (**f**, **h**). Example of the western blot used for protein quantification by densitometry after normalization with the level of βIII-tubulin (**i**). Immediately after the conditioning session animals received an injection of either vehicle (Veh; NaCl 0.9% i.p., white symbol) or Cort (2 mg/kg i.p., black symbol). Gray symbol: control animals that were manipulated but not exposed to conditioning. Magnitude of tone conditioning represented by a normalized ratio: (tone − ((pre + post)/2))/(tone + ((pre + post)/2)) (**b**). Student’s *t* test and Sidak’s and Bonferroni/Dunn’s multiple comparisons post hoc test after ANOVA: *p < 0.05, **p < 0.01, ***p < 0.005 compared to Veh group. Plotted values are means ± sem.

**Fig. 4:**
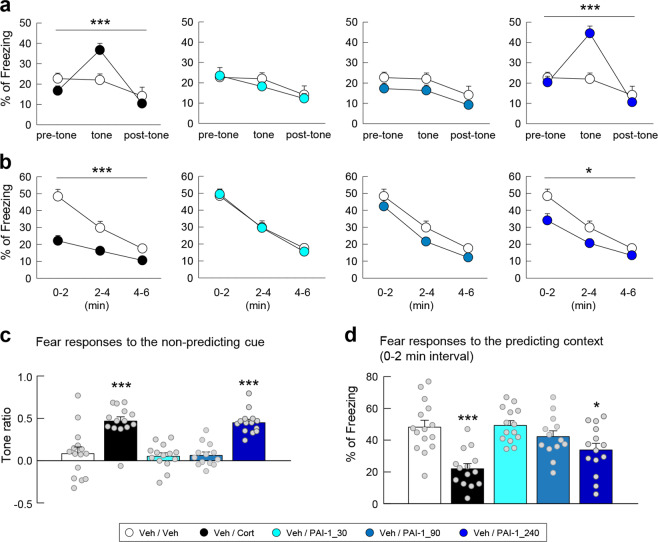
Effects of different doses of PAI-1 on PTSD-like memory. Fear responses, expressed as percent of time spent freezing, 24 h after conditioning in C57BL/6J mice exposed in a safe environment to the tone not predicting the threat (nonpredicting cue, **a** and **c**) or to the environment in which the conditioning was performed (predicting context, **b** and **d**). Immediately after the conditioning session animals received one of the following treatments: an injection of vehicle (Veh; NaCl 0.9% i.p., white symbol); an injection of Cort (2 mg/kg i.p., black symbol); an intrahippocampal infusion of PAI-1 (30, 90, or 240 ng/side, symbols with different shadows of blue). Magnitude of tone conditioning represented by a normalized ratio: (tone − ((pre + post)/2))/(tone + ((pre + post)/2)) (**c**). Bonferroni/Dunn’s multiple comparisons post hoc test after ANOVA: *p < 0.05, ***p < 0.005 compared to Veh/Veh group. Plotted values are means ± sem.

**Fig. 5:**
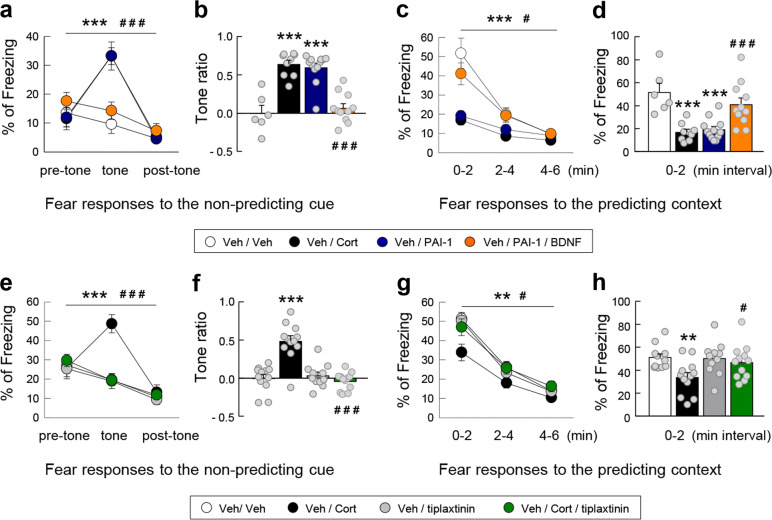
The increase in PAI-1 is a sufficient and necessary condition for the induction of PTSD-like memory. Fear responses, expressed as percent of time spent freezing, 24 h after conditioning in C57BL/6J mice exposed in a safe environment to the tone not predicting the threat (nonpredicting cue, **a**, **b**, **e**, and **f**) or to the environment in which the conditioning was performed (predicting context, **c**, **d**, **g**, and **h**). Immediately after the conditioning session animals received one of the following treatments: an injection of (white symbol) vehicle (Veh; NaCl 0.9% i.p.) alone or in combination with the intrahippocampal infusion of either (blue symbol) PAI-1 (240 ng/side) or (orange symbol) PAI-1 (240 ng/side) + mature BDNF (100 ng/side); an injection of (black symbol) Cort alone (2 mg/kg i.p.) or in combination with the intrahippocampal infusion of either the PAI-1 antagonist tiplaxtinin (5 ng/side, green symbol) or the vehicle of tiplaxtinin (gray symbol). Magnitude of tone conditioning represented by a normalized ratio: (tone − ((pre + post)/2))/(tone + ((pre + post)/2)) (**b** and **f**). Bonferroni/Dunn’s multiple comparisons post hoc test after ANOVA: **p < 0.001 and ***p < 0.005 vs Veh/Veh group, ^#^*p* < 0.05 and ^###^*p* < 0.005 compared to Veh/PAI-1 and Cort groups. Plotted values are means ± sem.

Male C57BL/6J mice [*n* = 19 in experiment 1 (Fig. 3a–d), *n* = 70 in experiment 2 (Fig. 4), *n* = 37 in experiment 3 (Fig. 5a–d), and *n* = 47 in experiment 4 (Fig. 5e–h)] 3–4 months old (Charles River Laboratory, France) were used. Mice were surgically implanted bilaterally 1 mm above the dorsal hippocampus (A/P, −2 mm; M/L, ±1.3 mm; D/V, 0.9 mm; relative to dura and bregma) following Franklin and Paxinos’s mouse brain atlas [46] then allowed to recover for 8 days before the behavioral experiments.

##### Adaptive vs maladaptive (PTSD-like) fear memory

The behavioral model based on a general fear conditioning procedure has been fully described in a previous study [7].

1. Pre-exposure: The day before fear conditioning, each mouse was placed individually in an opaque PVC chamber (30 × 24 × 22 cm) with an opaque PVC floor, for 2 min, in a brightness of 10 lux. This pre-exposure allowed the mice to acclimate and become familiar with the chamber used for the cue alone test (“safe context”).
2. Induction of adaptive vs PTSD-like fear memory: Acquisition of fear conditioning was performed in a different context, consisting in a Plexiglas conditioning chamber (30 × 24 × 22 cm) with the floor connected to a shock generator, in a brightness of 110 lux, giving access to the different visual-spatial cues in the experimental room. Briefly, each animal placed in the conditioning chamber for 4 min received two footshocks (0.4 mA, 50 Hz, 1 s), which never co-occurred with two tone deliveries (70 dB, 1 kHz, 15 s). This tone-shock unpairing paradigm is known to make the contextual cues the primary stimuli that become associated with the footshock [7, 47]. Consequently, the phasic tone, although salient, is not predictive of the shock delivery, whereas the static contextual cues constitute the main predictor of the shock. Immediately after the acquisition of fear conditioning, mice received a systemic injection of either NaCl or Cort (see below for details). An adaptive fear memory will therefore be attested in control (NaCl injected) mice by the expression of highly conditioned fear when re-exposed to the conditioning context and no conditioned fear when re-exposed to the irrelevant tone cue (in the safe context). In contrast, Cort-injected mice will display a maladaptive (PTSD-like) memory attested by an abnormally high fear response to the irrelevant tone (cue-based hypermnesia) together with a decreased conditioned fear to the conditioning context (contextual amnesia) [7].
3. Memory tests: After fear conditioning, each animal was returned to its home cage and 24 h later, all mice were submitted to two memory tests during which freezing behavior, defined as a lack of any movement except for respiratory-related movements, was measured and used as an index of conditioned fear. During these two memory tests, animals were continuously recorded for off-line second-by-second scoring of freezing by an observer blind to the experimental groups. Mice were first re-exposed to the tone within the “safe” context during which three successive recording sessions of the behavioral responses were performed: one before (first 2 min), one during (next 2 min), and one after (last 2 min) tone presentation. Conditioned response to the tone is expressed by the percentage of freezing during the tone presentation compared to the levels of freezing expressed before and after tone presentation (repeated measures on three blocks of freezing). The strength and specificity of this conditioned fear is attested by a ratio that represents the increase in the percentage of freezing with the tone with respect to a baseline freezing level [i.e., pre- and post-tone periods mean: (tone − ((pre + post)/2))/(tone + ((pre + post)/2))]. Indeed, a conditioned fear specific to the discrete tone CS implies a lower level of freezing when the shock is not expected (i.e., before and after the tone presentation) compared to the freezing level expressed during the tone presentation (high ratio value). Two hours later, mice were re-exposed to the conditioning context alone for 6 min (without the tone cue). Freezing to the context was calculated as the percentage of the total time spent freezing during the successive three blocks of 2-min periods of the test. While the first block is the critical block attesting difference between animals that are conditioned to the conditioning context and those that are not or less conditioned, the following two blocks are presented in order to assess a gradual extinction of the fear responses in the absence of shock.
4. Molecular analysis: In the experiment (Fig. 3e–i and Fig. S2) measuring the modulation of GC-mediated PAI-1 expression and Erk1/2^MAPK^ signaling pathway after the acquisition of fear conditioning, separate groups (*n* = 6–7 per group) of C57/BL6J mice were sacrificed 1, 2, 3, 6, and 24 h after the acquisition of fear conditioning and Cort or vehicle (NaCl 0.9%) injection. Naive C57/BL6J mice (*n* = 15) were used to quantify basal protein expression levels. Dorsal hippocampi were then collected and assayed for immunoblotting analysis.

##### Drug injections

Immediately after the acquisition of fear conditioning, mice were randomly divided into groups firstly according to first their systemic injection of Cort and secondly their specific intrahippocampal infusion. Cort (2 mg/kg in a volume of 0.1 ml/10 g body weight) or vehicle (NaCl 0.9%) was administered i.p [7], while PAI-1, mature BDNF and tiplaxtinin were intrahippocampally infused.

Experiment 1 (Fig. 3a–d): (1) Vehicle (NaCl 0.9%, *n* = 8). (2) Cort (2 mg/kg, *n* = 11).

Experiment 2 (Fig. 4): (1) Vehicles (artificial CSF (aCSF) + NaCl 0.9%, *n* = 15). (2) Cort (2 mg/kg + aCSF, *n* = 14), PAI-1 (30 ng/side + NaCl 0.9%, *n* = 14), PAI-1 (90 ng/side + NaCl 0.9%, *n* = 13), and PAI-1 (240 ng/side + NaCl 0.9%, *n* = 14).

Experiment 3 (Fig. 5a–d): (1) Vehicles (aCSF + NaCl 0.9%, *n* = 6). (2) Cort (2 mg/kg + aCSF, *n* = 8), PAI-1 (240 ng/side + NaCl 0.9%, *n* = 12), and PAI-1 + mature BDNF (240 ng/side + 100 ng/side, respectively, + NaCl 0.9%, *n* = 11).

Experiment 4 (Fig. 5e–h): (1) Vehicles (aCSF + dimethyl sulfoxide (DMSO), *n* = 12). (2) Cort (2 mg/kg + DMSO, *n* = 12), tiplaxtinin (5 ng/side + NaCl 0.9%, *n* = 11), and tiplaxtinin + Cort (5 ng/side + 2 mg/kg, respectively, *n* = 12).

PAI-1 and BDNF were diluted in aCSF and tiplaxtinin in 1.6% DMSO and then diluted in aCSF. Bilateral infusions of 0.3 μl/side were administered into the dorsal hippocampus immediately after acquisition of fear conditioning at a constant rate (0.1 µl/min).

##### Histology

A detailed description of the histological protocol was reported previously [26, 27]. Briefly, after completion of the behavioral study, animals were sacrificed in order to evaluate the cannulae placements.

### Data analysis

All experiments involving mice and rats were performed according to the protocols approved by the Aquitaine-Poitou Charentes local ethics committee (authorization number APAFlS#7397-20161 02814453778 v2) in strict compliance with the French Ministry of Agriculture and Fisheries (authorization number D33-063-096) and European Union Council Directive (2010/63/EU). All efforts were made to minimize animal suffering and to reduce the number of rodents used, while maintaining reliable statistics. All experiments were conducted with experimenters blind to drug treatment conditions; no randomization method for the constitution of the experimental groups was applied. The sample size was chosen to ensure adequate statistical power for all experiments. Differences in the areas under the curve were analyzed using two-tailed unpaired Student’s *t* test. For the qPCR and western blot experiments on PC12 cells, dorsal hippocampal slices of Sprague-Dawley rats and stressed C57BL/6J mice, data were analyzed by one-way analysis of variance (ANOVA) followed, when results were significant, by Dunnett’s multiple comparisons post hoc test. For the western blot experiments on “PTSD” C57BL/6J mice, data were analyzed by two-way ANOVA followed, when results were significant, by Sidak’s multiple comparisons post hoc test using treatment (Veh vs Cort) and time as main factors. For the behavioral experiments, statistical analyses were performed using ANOVA followed by Bonferroni/Dunn post hoc test for pairwise comparisons. All values were expressed as mean ± s.e.m. A significance level of *p* < 0.05 was used for all statistical analyses. Statistical significance was expressed as *p < 0.05; **p < 0.01; ***p < 0.005 vs Veh group or basal conditions and ^#^*p* < 0.05; ^###^*p* < 0.005 vs Cort or PAI-1 groups.

## Results

### Cort and stress stimulated the expression of PAI-1 protein

Our previous reports demonstrated that the tPA/plasmin system induced by GC-activated GR is a core effector in the regulation of the pro-BDNF/BDNF balance allowing, through the activation of the TrkB/Erk1/2^MAPK^ signaling cascade, the formation of normal fear memory [26*–*28]. Interestingly, the major potential physiological inhibitor of this pro-memory cascade, the tPA inhibitor PAI-1, also displays glucocorticoid responsive elements (GRE) in the regulatory sequences of its gene [48]. Since the effects of GC and stress on PAI-1 are unknown, in a first experiment we treated PC12 cells, which expresses both endogenous GR and PAI-1 [26, 49], with Cort the major GC in rodents. After 3 h of treatment, Cort (100 and 1000 nM) strongly increased the expression of PAI-1 mRNA (Fig. 1a; *F*_2,15_ = 100.7, *p* < 0.0001) and protein (Fig. 1b, c; *F*_2,13_ = 19.03, *p* = 0.0001 and Fig. S1a, b). In a second experiment, we then assessed the expression of PAI-1 in the hippocampus [50], the major target of the GC effect on memory (Fig. 1d, e and Fig. S1c, d). In hippocampal slices, concentrations of Cort (10 nM), mimicking moderate stress conditions, first induced an increase in tPA (*F*_2,15_ = 4.942, *p* = 0.0224), P-TrkB (*F*_2,14_ = 11.41, *p* = 0.0011), and P-Erk1/2^MAPK^ (*F*_2,14_ = 4.663, *p* = 0.0281) (1 h after treatment) followed (3 h after treatment) by an increase in PAI-1 (*F*_2,13_ = 7.612, *p* = 0.0065). The increase in PAI-1 at 3 h was associated with the return of tPA, P-TrkB, and P-Erk1/2^MAPK^ at basal levels (Fig. 1d, e). In contrast, high concentrations of Cort (1000 nM) induced an early increase in PAI-1 (*F*_2,13_ = 5.497, *p* = 0.0186) (1 h after treatment) which was also accompanied by the suppression of the increase in memory-promoting proteins tPA (*F*_2,15_ = 5.599, *p* = 0.0153; Veh vs Cort *p* = 0.5450), P-TrkB (*F*_2,14_ = 0.100, *p* = 0.9050), and P-Erk1/2^MAPK^ (*F*_2,14_ = 0.5634, *p* = 0.5817) after 3 h treatment (Fig. 1d, e). Combining in vitro and ex vivo approaches, GR-expressing cell lines and hippocampal slices, respectively, we identified PAI-1 as a plausible upstream molecular effector activated by increasing amounts of GC. Since the secretion of Cort increases systemically in response to stress [41], in a third experiment we studied the effects of different stress intensities on the expression of tPA and PAI-1 in the hippocampus of C57BL/6J mice. We compared 30 min, 1 h, and 3 h of restraint stress which induces progressively higher plasma levels of Cort (Fig. 2a; *t* = 13.26, df = 10 and *F*_2,15_ = 159.1, *p* < 0.0001). Strikingly after 30 min of moderate stress only the memory-promoting proteins tPA (*F*_3,14_ = 4.988, *p* = 0.0147; base vs 30 min *p* = 0.0052) and P-Erk1/2^MAPK^ (*F*_3,14_ = 21.05, *p* < 0.0001; base vs 30 min *p* = 0.0118) were increased (Fig. 2b, c and Fig. S1e, f). In contrast, in more intense stress conditions (1 and 3 h) there was a strong increase in PAI-1 (*F*_3,14_ = 4.973, *p* = 0.0148; base vs 60 min *p* = 0.0423 and base vs 180 min *p* = 0.0223) associated with the inhibition of P-Erk1/2^MAPK^ (*F*_3,14_ = 21.05, *p* < 0.0001; base vs 60 min *p* = 0.0421 and base vs 180 min *p* = 0.0054) which gradually went below basal levels (Fig. 2b, c). The results of this first series of experiments suggest that a moderate increase in GC concentrations during stress first triggers the activation of the adaptive memory-promoting tPA/TrkB/Erk1/2^MAPK^ signaling cascade and subsequently of its inhibitor PAI-1. However, during intense stress a high level of GC induces early activation of PAI-1 which inhibits the adaptive memory-promoting tPA/TrkB/Erk1/2^MAPK^ signaling cascade.

### Increased expression of PAI-1 protein is a sufficient condition to induce PTSD-like memories

In a second series of experiments, we investigated whether changes in the expression of PAI-1 could determine the appearance of PTSD-like memories, which were evaluated using a previously described mouse model [7]. Mice were submitted to a threatening situation—the delivery of an electric footshock—when exposed to a specific context (conditioning cage). A discrete cue (a tone) was also repeatedly presented during conditioning but was never paired with shock delivery. In these conditions, the context is the correct predictor of the threat (predicting context), whilst the cue although present with the threat does not predict it (nonpredicting cue). Twenty-four hours after this conditioning procedure, animals were re-exposed first to the cue alone in a familiar and safe environment and then to the conditioning context without the cue [7]. As previously observed [7], vehicle-injected mice (*n* = 8) showed no fear response (no freezing) to the irrelevant (nonpredictive) tone (Fig. 3a, b) and high fear response to the context, predictive of the shock (Fig. 3c, d). However, if mice were injected with Cort (2 mg/kg) immediately after conditioning, PTSD-like memory impairments appeared. PTSD-like memory in Cort-injected mice (*n* = 11) was attested by an abnormally high fear response to the tone (treatment × block during the tone test: *F*_2,34_ = 4.278; *p* = 0.022; treatment effect in tone ratio: *p* = 0.0452), and a decreased fear to the context [treatment effect (three blocks) during the context test: *F*_1,34_ = 4.599; *p* = 0.0467], which was specifically observed during the first 2-min block (*p* = 0.0055). Like PTSD patients, mice injected with Cort lost the ability to restrict fear to the appropriate situation or cue [13]. In addition, we showed previously that postconditioning restraint stress mimics the effects of exogenous administration of Cort on fear memory, producing PTSD-like memory after a relatively high stressful situation [7]. Using this behavioral model, we first compared the expression of P-Erk1/2^MAPK^ and PAI-1 in the dorsal hippocampus (Fig. 3e–i and Fig. S2). In control mice, showing normal fear memory, the concentrations of the adaptive memory-promoting proteins P-Erk1/2^MAPK^ progressively increased after the conditioning session, whilst it decreased to below basal levels in animals that developed PTSD-like memories (Fig. 3e, i; interaction time × treatment: *F*_4,40_ = 1.375, *p* = 0.2598; time: *F*_4,40_ = 2.988, *p* = 0.0300; treatment: *F*_1,10_ = 14.96, *p* = 0.0031) as shown by the analysis of the area under the curve (Fig. 3f; *t* = 5.362, df = 10, *p* = 0.0003). The opposite pattern was observed for PAI-1 which reached much higher concentrations in animals showing PTSD-like memories than in control mice (Fig. 3g, i; interaction time × treatment: *F*_4,48_ = 2.648, *p* = 0.0446; time: *F*_4,48_ = 8.863, *p* < 0.0001; treatment: *F*_1,12_ = 25.89, *p* = 0.0003) as also shown by the analysis of the area under the curve (Fig. 3h; *t* = 6.835, df = 12, *p* < 0.0001). In a second experiment, we assessed whether this increase in PAI-1 expression was a sufficient condition for inducing PTSD-like memories. For this purpose, after conditioning, we injected different concentrations of PAI-1 into the dorsal hippocampus (Fig. 4). Similarly to what was observed after Cort, intrahippocampal injection of PAI-1 at the highest dose (240 ng/side) induced PTSD-like memory impairments. Indeed, compared to vehicle-injected mice (*n* = 15), PAI-1-injected mice (*n* = 14) displayed PTSD-like fear memory with an abnormally high fear response to the salient, but irrelevant, tone (Fig. 4a, c—treatment × block during the tone test: *F*_2,54_ = 20.709; *p* < 0.0001; treatment effect in tone ratio: *p* < 0.0001), and a decreased fear response to the predictive context (Fig. 4b, d—treatment effect (three blocks) during the context test: *F*_1,54_ = 5.006; *p* = 0.0337), which was mainly observed during the first 2-min block (*p* = 0.0202). When lower doses were used (30 and 90 ng), PAI-1-injected mice did not differ from vehicle-injected mice whatever the memory test considered (tone test: all *P* < 0.541, *p* = ns; context test: all *F* < 2.66, *p* = ns). The results of this second series of experiments indicate that the increase in PAI-1 expression triggered by GC is a sufficient condition to induce PTSD-like memories.

### Increased expression of PAI-1 protein is a necessary condition to induce PTSD-like memories

In the third series of experiments, we wanted to establish whether it was possible to block the development of PTSD-like memory. To address this issue, we first assessed the hypothesis whether PTSD-like memory induced by injection of PAI-1 (Fig. 4) is blocked by infusion of mature BDNF in the dorsal hippocampus. Mature BDNF should be sufficient to bypass the PAI-1 inhibitory effect on the tPA/plasmin system to allow normal fear memory. We found that PAI-1-induced PTSD-like memory formation was completely blocked by the concomitant injection of mature BDNF that normalized fear memory (Fig. 5a–d). While PAI-1-injected mice (*n* = 12), like Cort-injected mice (*n* = 8), displayed an abnormally high fear response to the tone (treatment × block in tone test for PAI-1 vs Veh: *F*_2,32_ = 15.946; *p* < 0.0001 and for Cort vs Veh: *F*_2,26_ = 25.331; *p* < 0.0001; tone ratio: both *p* < 0.0001), PAI-1/BDNF-injected mice (*n* = 11) did not display any fear response to the irrelevant tone (treatment × block for PAI-1/BDNF vs PAI-1: *F*_2,42_ = 26.391; *p* < 0.0001; tone ratio: *p* < 0.0001), and thus did not differ from control (Veh-injected) mice (Fig. 5a, b). In parallel, while PAI-1-injected mice, like Cort-injected mice, displayed a decreased fear response to the context when compared to control (treatment effect in context test for PAI-1 vs Veh: *F*_1,32_ = 11.269; *p* = 0.0040, and for Cort vs Veh: *F*_1,26_ = 19.848; *p* = 0.0006; first 2-min block: both *p* ≤ 0.0003), PAI-1/BDNF-injected mice displayed a normally high fear response to the predictive context [treatment effect for PAI-1/BDNF vs PAI-1: *F*_1,42_ = 5.703; *p* = 0.0264], which was mainly observed during the first 2-min block (*p* = 0.0019) and did not differ from vehicle-injected mice (Fig. 5c, d). These data indicate that PAI-1 likely induces PTSD-like memory by blocking the tPA-mediated proteolytic processing of pro-BDNF to mature BDNF [51, 52]. This evidence suggests that inhibiting hippocampal PAI-1 could be a valuable therapeutic strategy for the treatment of PTSD-like memory. Among the several PAI-1 inhibitors, tiplaxtinin (PAI-039) has been well characterized in several animal models, showing promise as a PAI-1 antagonist [53*–*55]. In this experiment, we assessed whether an increase in PAI-1 expression was a necessary condition for the appearance of PTSD-like memories. We demonstrated that intrahippocampal inhibition of PAI-1 by the injection of its antagonist tiplaxtinin (PAI-039) immediately after the conditioning session prevented PTSD-like memory formation in Cort-treated animals (Fig. 5e–h). Indeed, tiplaxtinin injection prevented both the Cort-induced increased fear response to the irrelevant tone (Fig. 5e, f—treatment × block in tone test for Cort vs Veh: *F*_2,44_ = 21.693; *p* < 0.0001, and for Cort/tiplaxtinin vs Cort: *F*_2,44_ = 24.719; *p* < 0.0001; tone ratio: both *p* < 0.0001), and the Cort-induced decreased fear response to the predictive context (Fig. 5g, h—treatment effect in context test for Cort vs Veh: *F*_1,44_ = 8.946; *p* = 0.0067, and for Cort/tiplaxtinin vs Cort: *F*_1,44_ = 5.293; *p* = 0.0313), which was mainly observed during the first 2-min block (*p* = 0.0425).

Taken together, the findings of these experiments indicated that an increase in PAI-1 levels in the hippocampus triggered by high levels of GC is a sufficient and necessary condition to induce PTSD-like memories.

## Discussion

Uncovering the molecular mechanisms of the shift from beneficial to harmful effects of stress and GC is a key question particularly relevant to understand the pathophysiological mechanism through which life events can induce psychiatric disorders. While previous studies have demonstrated differential, and even opposite, effects of stress on cognitive processes [24, 56–59], the present findings identify a key molecular signaling pathway, which in the dorsal hippocampus, underlies such dissociation (i.e., beneficial vs harmful effects of stress and GC) notably in the formation of normal (adaptive) vs maladaptive (PTSD-like) fear memory.

**Fig. 6:**
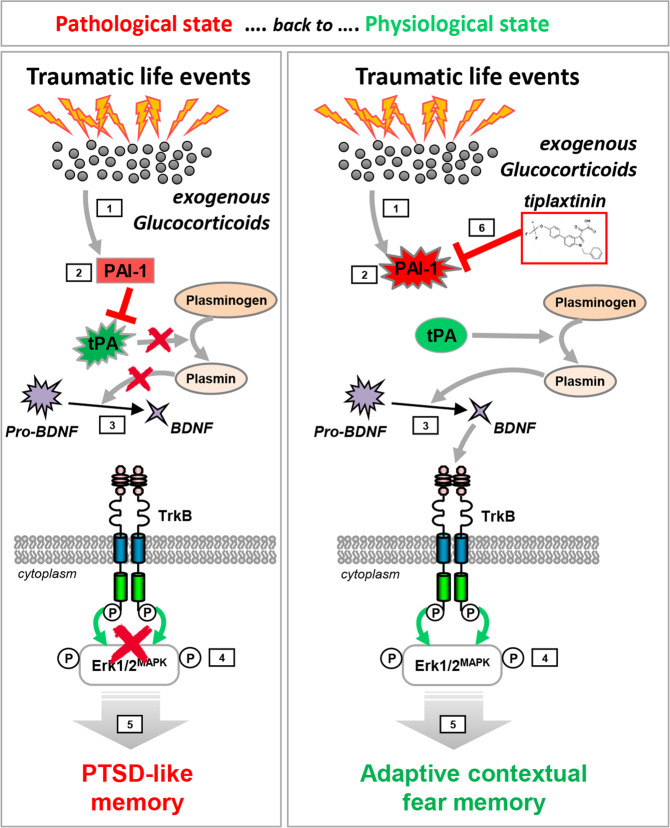
Schematic model of glucocorticoid (GC)-induced molecular mechanism mediating the shift from adaptive to PTSD-like memory. Under traumatic conditions, high levels of GC [1] induce the expression of PAI-1 [2] that by inhibiting tPA blocks the proteolytic processing of pro-BDNF in mature BDNF [3] by plasmin. The resulting consecutive blockade of TrkB/Erk1/2^MAPK^ activation [4] leads to impairment of hippocampal function, which thereby promotes the formation of a decontextualized (cue based) traumatic memory [5]. By lowering hippocampal PAI-1 activity, tiplaxtinin [6] restores the activity of this molecular cascade, promotes the formation of a hippocampal-dependent adaptive (“contextualized”) fear memory and thus normalizes traumatic memory. This model, based on several studies including our own, is discussed in the main text.

We have previously shown that in moderate stress conditions, GC hormones induce the expression of the tPA protein which by increasing the production of mature BDNF triggers the activation of the TrkB/Erk1/2^MAPK^ cascade which strengthens the memory trace of the stress-related event [26*–*28]. Here, we showed that the activity of the tPA/BDNF/TrkB/Erk1/2^MAPK^ cascade is then inhibited by the delayed production of the tPA inhibitor PAI-1. However, in the case of particularly stressful conditions and very high levels of GC, the production of PAI-1 is triggered early on. PAI-1 then blocks the activity of tPA and inhibits the promnesic BDNF/TrkB/Erk1/2^MAPK^ signaling cascade, inducing PTSD-like memory. By lowering hippocampal PAI-1 activity, tiplaxtinin [53*–*55] restored the formation of a hippocampal-dependent adaptive (“contextualized”) fear memory and thus normalizes traumatic memory (Fig. 6).

Several lines of evidence support the involvement of impairment of BDNF processing mediated by PAI-1 in the pathophysiology of stress-related diseases. Firstly, impaired BDNF function has been associated with PTSD both in rodents and human [60]. Secondly, PTSD patients have a higher risk of cardiovascular pathophysiologies and notably atherothrombosis [61], for which high levels of PAI-1 is a known risk factor [62]. Thirdly, elevated PAI-1 levels have also been observed in patients with major depressive disorders, another stress-induced condition [63*–*65].

An increase in PAI-1 could mediate the pathological effects of stress not only by decreasing the production of mature BDNF but also by promoting the accumulation of pro-BDNF that is no longer cleaved into mature BDNF by the tPA-activated plasmin. Indeed, although long considered to be inactive, pro-BDNF forms are able to form a ternary complex with the p75^NTR^ and sortilin receptors, to induce neuronal cell death by apoptosis [66]. In addition, pro-BDNF/p75^NTR^ signaling has been shown to have the opposite effect on synaptic plasticity, inducing LTD, whilst BDNF/TrkB signaling induces LTP [67]. These results are consistent with brain imaging findings showing hippocampal atrophy reported in PTSD subjects [19, 35]. Although not systematically observed in PTSD subjects [36], hippocampal atrophy could be both a consequence of an extreme stress-induced PTSD and a risk factor for this pathology [35]. In addition, the present data identifying a key molecular mechanism in the dorsal hippocampus involved in the formation of normal vs pathological (PTSD-like) fear memory are in full compliance with our recent findings showing that inhibiting dorsal CA1 (dCA1) neurons during stress produces PTSD-like fear memory, whereas activating dCA1 neurons in traumatic conditions prevents PTSD-like memory formation [21].

Although the biphasic effects of activated GR have been described before in other contexts [68, 69], the exact mechanism through which the dose-dependent effects of GC regulate the transcription of the PAI-1 encoding gene deserves further discussion. Thus, GC effectiveness is determined by several selective tissue- and/or cell-specific components such as GC bioavailability dependent on metabolizing enzymes notably expression of 11beta-hydroxysteroid dehydrogenase, GR splice variants, contents in GR interacting proteins, and chromatin accessibility [24]. The PAI-1 promoter is known to have, in addition to GRE, response elements for the AP-1 transcription factor complex (Fos:Jun) [70]. A plausible explanation of the observed dose-dependent effects is that when moderate levels of activated GR are produced, GR/AP-1 heterodimers, which are known to promote reciprocal transcriptional interference, are mostly formed and prevent PAI-1 transcription through a protein–protein interaction mediated-sequestration process [71, 72]. In contrast, when high levels of activated GR are produced, for example, after intense stress, GR/GR homodimers are now formed which are able to activate the transcription of the PAI-1 encoding gene. Another hypothesis is the presence of a canonical E-Box motif within the mouse PAI-1 promoter responsive to dexamethasone that showed to drive rhythmic mouse PAI-1 transcription in vitro [73]. This E-Box motif could act as a low affinity binding site activated only by high levels of GC-activated GR, through direct or indirect mechanisms, acting as a “rheostat” to modify the intensity of the GC response.

The PTSD-like phenotype relies on the systemic injection of Cort combined to fear conditioning [7, 21]. Nevertheless it has to be taken into account that the exogenous Cort in the blood may impact the endogenous negative feedback loop by attenuating the increase in circulating free baseline Cort endogenous levels over total plasma levels, which may modify the subsequent behavioral effect of Cort. Therefore, amount of exogenous over endogenous Cort is worth considering although we showed previously that postconditioning restraint stress was able to mimick the effects of an exogenous administration of Cort, producing PTSD-like memory impairments [7].

Our data confirm that the systemic injection of Cort immediately after fear conditioning is sufficient to induce PTSD-like memory impairments [7, 21]. However, GC hormones as the end product of the HPA axis activity are not the only biological components of the stress response [74]. In addition to neuroendocrine response, the release of catecholamines, in particular norepinephrine (NE), by the locus coeruleus (LC) was shown to play a meaningful role in the pathophysiology of PTSD [75, 76]. Interestingly, corticotropin-releasing hormone (CRH), as a major integrator of the stress response, was also shown to activate the NE-LC system [77, 78] and to promote tPA activity in the amygdala to elicit subsequent anxiety-related behavioral effects [79, 80]. Therefore, these interconnected systems through an increase of CRH bidirectional signaling in NE-LC and amygdalar neurons and a decrease of GC-mediated hippocampal signaling could act synergistically to coordinate the expression of PTSD symptoms.

In conclusion, our data show that the transition from adaptive to maladaptive stress-related hippocampal-dependent memories is mediated by a shift in balance between tPA and PAI-1 proteins, with an adaptive increase in memory appearing when the ratio is in favor of tPA [28] and PTSD-like memory when it shifts in favor of PAI-1. As a consequence, PAI-1 levels after a traumatic event could be a predictive biomarker of the appearance of PTSD and pharmacological inhibition of PAI-1 activity a new therapeutic approach to this debilitating condition.

## Supplementary information

Supp Figure S1

Supp Figure S2

SUPPLEMENTARY INFORMATION

## References

[1] McEwen BS (2000). The neurobiology of stress: from serendipity to clinical relevance. Brain Res.

[2] De Kloet ER, Joels M, Holsboer F (2005). Stress and the brain: from adaptation to disease. Nat Rev Neurosci.

[3] Finsterwald C, Alberini CM (2014). Stress and glucocorticoid receptor-dependent mechanisms in long-term memory: from adaptive responses to psychopathologies. Neurobiol Learn Mem.

[4] Piazza PV, Le Moal M (1998). The role of stress in drug self-administration. Trends Pharm Sci.

[5] Roozendaal B (2002). Stress and memory: opposing effects of glucocorticoids on memory consolidation and memory retrieval. Neurobiol Learn Mem.

[6] McGaugh JL, Roozendaal B (2002). Role of adrenal stress hormones in forming lasting memories in the brain. Curr Opin Neurobiol.

[7] Kaouane N, Porte Y, Vallee M, Brayda-Bruno L, Mons N, Calandreau L (2012). Glucocorticoids can induce PTSD-like memory impairments in mice. Science.

[8] Deppermann S, Storchak H, Fallgatter AJ, Ehlis AC (2014). Stress-induced neuroplasticity: (mal)adaptation to adverse life events in patients with PTSD-a critical overview. Neuroscience.

[9] American Psychiatric Association. (2013). Diagnostic and statistical manual of mental disorders (DSM–5).

[10] Kessler RC, Sonnega A, Bromet E, Hughes M, Nelson CB (1995). Posttraumatic stress disorder in the National Comorbidity Survey. Arch Gen Psychiatry.

[11] Yehuda R, Hoge CW, McFarlane AC, Vermetten E, Lanius RA, Nievergelt CM (2015). Post-traumatic stress disorder. Nat Rev Dis Prim.

[12] Breen MS, Tylee DS, Maihofer AX, Neylan TC, Mehta D, Binder EB (2018). PTSD blood transcriptome mega-analysis: shared inflammatory pathways across biological sex and modes of trauma. Neuropsychopharmacology.

[13] Desmedt A, Marighetto A, Piazza PV (2015). Abnormal fear memory as a model for posttraumatic stress disorder. Biol Psychiatry.

[14] Elzinga BM, Bremner JD (2002). Are the neural substrates of memory the final common pathway in posttraumatic stress disorder (PTSD)?. J Affect Disord.

[15] Layton B, Krikorian R (2002). Memory mechanisms in posttraumatic stress disorder. J Neuropsychiatry Clin Neurosci.

[16] Nutt DJ, Malizia AL (2004). Structural and functional brain changes in posttraumatic stress disorder. J Clin Psychiatry.

[17] Desmedt A, Marighetto A, Richter-Levin G, Calandreau L (2015). Adaptive emotional memory: the key hippocampal-amygdalar interaction. Stress.

[18] Desmedt A, Marighetto A, Piazza PV (2015). Abnormal fear memory as a model for posttraumatic stress disorder. Biol Psychiatry.

[19] Sala M, Perez J, Soloff P, Ucelli di NS, Caverzasi E, Soares JC (2004). Stress and hippocampal abnormalities in psychiatric disorders. Eur Neuropsychopharmacol.

[20] Rauch SL, Shin LM, Phelps EA (2006). Neurocircuitry models of posttraumatic stress disorder and extinction: human neuroimaging research-past, present, and future. Biol Psychiatry.

[21] Al Abed AS, Ducourneau EG, Bouarab C, Sellami A, Marighetto A, Desmedt A (2020). Preventing and treating PTSD-like memory by trauma contextualization. Nat Commun.

[22] Salehi B, Cordero MI, Sandi C (2010). Learning under stress: the inverted-U-shape function revisited. Learn Mem.

[23] Yerkes RM, Dodson JD (1908). The relation of strength of stimulus to rapidity of habit formation. J Comp Neurolog Psychol.

[24] Joels M (2006). Corticosteroid effects in the brain: U-shape it. Trends Pharm Sci.

[25] de Quervain D, Schwabe L, Roozendaal B (2017). Stress, glucocorticoids and memory: implications for treating fear-related disorders. Nat Rev Neurosci.

[26] Revest JM, Kaouane N, Mondin M, Le RA, Rouge-Pont F, Vallee M (2010). The enhancement of stress-related memory by glucocorticoids depends on synapsin-Ia/Ib. Mol Psychiatry.

[27] Revest JM, Di Blasi F, Kitchener P, Rouge-Pont F, Desmedt A, Turiault M (2005). The MAPK pathway and Egr-1 mediate stress-related behavioral effects of glucocorticoids. Nat Neurosci.

[28] Revest JM, Le Roux A, Roullot-Lacarriere V, Kaouane N, Vallee M, Kasanetz F (2014). BDNF-TrkB signaling through Erk1/2 MAPK phosphorylation mediates the enhancement of fear memory induced by glucocorticoids. Mol Psychiatry.

[29] Roozendaal B, Williams CL, McGaugh JL (1999). Glucocorticoid receptor activation in the rat nucleus of the solitary tract facilitates memory consolidation: involvement of the basolateral amygdala. Eur J Neurosci.

[30] Reul JM (2014). Making memories of stressful events: a journey along epigenetic, gene transcription, and signaling pathways. Front Psychiatry.

[31] Gutièrrez-Mecinas, Trollope A, Collins, Morfett, Hesketh S, Kersanté (2011). Long-lasting behavioral responses to stress involve a direct interaction of glucocorticoid receptors with ERK1/2-MSK1-Elk-1 signaling. Proc Natl Acad Sci USA.

[32] Yehuda R, Boisoneau D, Lowy MT, Giller EL (1995). Dose-response changes in plasma cortisol and lymphocyte glucocorticoid receptors following dexamethasone administration in combat veterans with and without posttraumatic stress disorder. Arch Gen Psychiatry.

[33] Finsterwald C, Steinmetz AB, Travaglia A, Alberini CM (2015). From memory impairment to posttraumatic stress disorder-like phenotypes: the critical role of an unpredictable second traumatic experience. J Neurosci.

[34] Weikum ER, Knuesel MT, Ortlund EA, Yamamoto KR (2017). Glucocorticoid receptor control of transcription: precision and plasticity via allostery. Nat Rev Mol Cell Biol.

[35] Gilbertson MW, Shenton ME, Ciszewski A, Kasai K, Lasko NB, Orr SP (2002). Smaller hippocampal volume predicts pathologic vulnerability to psychological trauma. Nat Neurosci.

[36] Neylan TC, Lenoci M, Rothlind J, Metzler TJ, Schuff N, Du AT (2004). Attention, learning, and memory in posttraumatic stress disorder. J Trauma Stress.

[37] Nagai T, Kamei H, Ito M, Hashimoto K, Takuma K, Nabeshima T (2005). Modification by the tissue plasminogen activator-plasmin system of morphine-induced dopamine release and hyperlocomotion, but not anti-nociceptive effect in mice. J Neurochem.

[38] Kasanetz F, Lafourcade M, Deroche-Gamonet V, Revest JM, Berson N, Balado E (2013). Prefrontal synaptic markers of cocaine addiction-like behavior in rats. Mol Psychiatry.

[39] Diamond DM, Bennett MC, Fleshner M, Rose GM (1992). Inverted-U relationship between the level of peripheral corticosterone and the magnitude of hippocampal primed burst potentiation. Hippocampus.

[40] Joëls M, De Kloet ER (1994). Mineralocorticoid and glucocorticoid receptors in the brain. Implications for ion permeability and transmitter systems. Prog Neurobiol.

[41] Kitchener P, Di Blasi F, Borrelli E, Piazza PV (2004). Differences between brain structures in nuclear translocation and DNA binding of the glucocorticoid receptor during stress and the circadian cycle. Eur J Neurosci.

[42] Sarrazin N, Di Blasi F, Roullot-Lacarriere V, Rouge-Pont F, Le Roux A, Costet P (2009). Transcriptional effects of glucocorticoid receptors in the dentate gyrus increase anxiety-related behaviors. PLoS ONE.

[43] Chomczynski P, Sacchi N (1987). Single-step method of RNA isolation by acid guanidinium thiocyanate-phenol-chloroform extraction. Anal Biochem.

[44] Bustin SA, Benes V, Garson JA, Hellemans J, Huggett J, Kubista M (2009). The MIQE guidelines: minimum information for publication of quantitative real-time PCR experiments. Clin Chem.

[45] Livak KJ, Schmittgen TD (2001). Analysis of relative gene expression data using real-time quantitative PCR and the 2(-Delta Delta C(T)) method. Methods.

[46] Paxinos G, Franklin KBJ. The mouse brain in stereotaxic coordinates. 2nd ed. San Diego: Academic Press; 2001.

[47] Calandreau L, Trifilieff P, Mons N, Costes L, Marien M, Marighetto A (2006). Extracellular hippocampal acetylcholine level controls amygdala function and promotes adaptive conditioned emotional response. J Neurosci.

[48] Bruzdzinski CJ, Johnson MR, Goble CA, Winograd SS, Gelehrter TD (1993). Mechanism of glucocorticoid induction of the rat plasminogen activator inhibitor-1 gene in HTC rat hepatoma cells: identification of cis-acting regulatory elements. Mol Endocrinol.

[49] Vician L, Basconcillo R, Herschman HR (1997). Identification of genes preferentially induced by nerve growth factor versus epidermal growth factor in PC12 pheochromocytoma cells by means of representational difference analysis. J Neurosci Res.

[50] Salles FJ, Strickland S (2002). Localization and regulation of the tissue plasminogen activator-plasmin system in the hippocampus. J Neurosci.

[51] Pang PT, Teng HK, Zaitsev E, Woo NT, Sakata K, Zhen S (2004). Cleavage of proBDNF by tPA/plasmin is essential for long-term hippocampal plasticity. Science.

[52] Pawlak R, Rao BS, Melchor JP, Chattarji S, McEwen B, Strickland S (2005). Tissue plasminogen activator and plasminogen mediate stress-induced decline of neuronal and cognitive functions in the mouse hippocampus. Proc Natl Acad Sci USA.

[53] Elokdah H, Abou-Gharbia M, Hennan JK, McFarlane G, Mugford CP, Krishnamurthy G (2004). Tiplaxtinin, a novel, orally efficacious inhibitor of plasminogen activator inhibitor-1: design, synthesis, and preclinical characterization. J Med Chem.

[54] Smith LH, Dixon JD, Stringham JR, Eren M, Elokdah H, Crandall DL (2006). Pivotal role of PAI-1 in a murine model of hepatic vein thrombosis. Blood.

[55] Gerenu G, Martisova E, Ferrero H, Carracedo M, Rantamaki T, Ramirez MJ (2017). Modulation of BDNF cleavage by plasminogen-activator inhibitor-1 contributes to Alzheimer’s neuropathology and cognitive deficits. Biochim Biophys Acta.

[56] McEwen BS (2019). What is the confusion with cortisol?. Chronic Stress.

[57] Meaney MJ (2001). Maternal care, gene expression, and the transmission of individual differences in stress reactivity across generations. Annu Rev Neurosci.

[58] Schwabe L, Joëls M, Roozendaal B, Wolf OT, Oitzl MS (2012). Stress effects on memory: an update and integration. Neurosci Biobehav Rev.

[59] Diamond DM, Campbell AM, Park CR, Halonen J, Zoladz PR (2007). The temporal dynamics model of emotional memory processing: a synthesis on the neurobiological basis of stress-induced amnesia, flashbulb and traumatic memories, and the Yerkes-Dodson law. Neural Plast.

[60] Stratta P, Sanita P, Bonanni RL, de CS, Angelucci A, Rossi R (2016). Clinical correlates of plasma brain-derived neurotrophic factor in post-traumatic stress disorder spectrum after a natural disaster. Psychiatry Res.

[61] Wentworth BA, Stein MB, Redwine LS, Xue Y, Taub PR, Clopton P (2013). Post-traumatic stress disorder: a fast track to premature cardiovascular disease?. Cardiol Rev.

[62] Vaughan DE (2005). PAI-1 and atherothrombosis. J Thromb Haemost.

[63] Savoy C, Van Lieshout RJ, Steiner M (2017). Is plasminogen activator inhibitor-1 a physiological bottleneck bridging major depressive disorder and cardiovascular disease?. Acta Physiol.

[64] Lahlou-Laforet K, Alhenc-Gelas M, Pornin M, Bydlowski S, Seigneur E, Benetos A (2006). Relation of depressive mood to plasminogen activator inhibitor, tissue plasminogen activator, and fibrinogen levels in patients with versus without coronary heart disease. Am J Cardiol.

[65] Eskandari F, Mistry S, Martinez PE, Torvik S, Kotila C, Sebring N (2005). Younger, premenopausal women with major depressive disorder have more abdominal fat and increased serum levels of prothrombotic factors: implications for greater cardiovascular risk. Metabolism.

[66] Teng HK, Teng KK, Lee R, Wright S, Tevar S, Almeida RD (2005). ProBDNF induces neuronal apoptosis via activation of a receptor complex of p75NTR and sortilin. J Neurosci.

[67] Woo NH, Teng HK, Siao CJ, Chiaruttini C, Pang PT, Milner TA (2005). Activation of p75(NTR) by proBDNF facilitates hippocampal long-term depression. Nat Neurosci.

[68] Yokoyama K, Hayashi M, Mogi C, Sasakawa Y, Watanabe G, Taya K (2008). Dose-dependent effects of a glucocorticoid on prolactin production. Endocr J.

[69] Shi J, Wang L, Zhang H, Jie Q, Li X, Shi Q (2015). Glucocorticoids: dose-related effects on osteoclast formation and function via reactive oxygen species and autophagy. Bone.

[70] Descheemaeker KA, Wyns S, Nelles L, Auwerx J, Ny T, Collen D (1992). Interaction of AP-1-, AP-2-, and Sp1-like proteins with two distinct sites in the upstream regulatory region of the plasminogen activator inhibitor-1 gene mediates the phorbol 12-myristate 13-acetate response. J Biol Chem.

[71] Petta I, Dejager L, Ballegeer M, Lievens S, Tavernier J, De BK (2016). The interactome of the glucocorticoid receptor and its influence on the actions of glucocorticoids in combatting inflammatory and infectious diseases. Microbiol Mol Biol Rev.

[72] Karin M, Chang L (2001). AP-1-glucocorticoid receptor crosstalk taken to a higher level. J Endocrinol.

[73] Singletary JH, Chan D, Samani NJ, Chong NW (2008). The canonical E-box motif: a target for glucocorticoid action that drives rhythmic mouse Pai-1 transcription in vitro. Gene.

[74] Chrousos GP (2009). Stress and disorders of the stress system. Nat Rev Endocrinol.

[75] O’Donnell T, Hegadoren KM, Coupland NC (2004). Noradrenergic mechanisms in the pathophysiology of post-traumatic stress disorder. Neuropsychobiology.

[76] Hendrickson RC, Raskind MA (2016). Noradrenergic dysregulation in the pathophysiology of PTSD. Exp Neurol.

[77] Kovacs KJ (2013). CRH: the link between hormonal-, metabolic- and behavioral responses to stress. J Chem Neuroanat.

[78] Valentino RJ, Van BE (2008). Convergent regulation of locus coeruleus activity as an adaptive response to stress. Eur J Pharm.

[79] Pawlak R, Magarinos AM, Melchor J, McEwen B, Strickland S (2003). Tissue plasminogen activator in the amygdala is critical for stress-induced anxiety-like behavior. Nat Neurosci.

[80] Matys T, Pawlak R, Matys E, Pavlides C, McEwen BS, Strickland S (2004). Tissue plasminogen activator promotes the effects of corticotropin-releasing factor on the amygdala and anxiety-like behavior. Proc Natl Acad Sci USA.

