# Supplementary figures and images for: PAI-1 protein is a key molecular effector in the transition from normal to PTSD-like fear memory
Source: Mol Psychiatry. 2021 Jan 28;26(9):4968–81. doi: 10.1038/s41380-021-01024-1 (PMC8589667; doi:10.1038/s41380-021-01024-1)

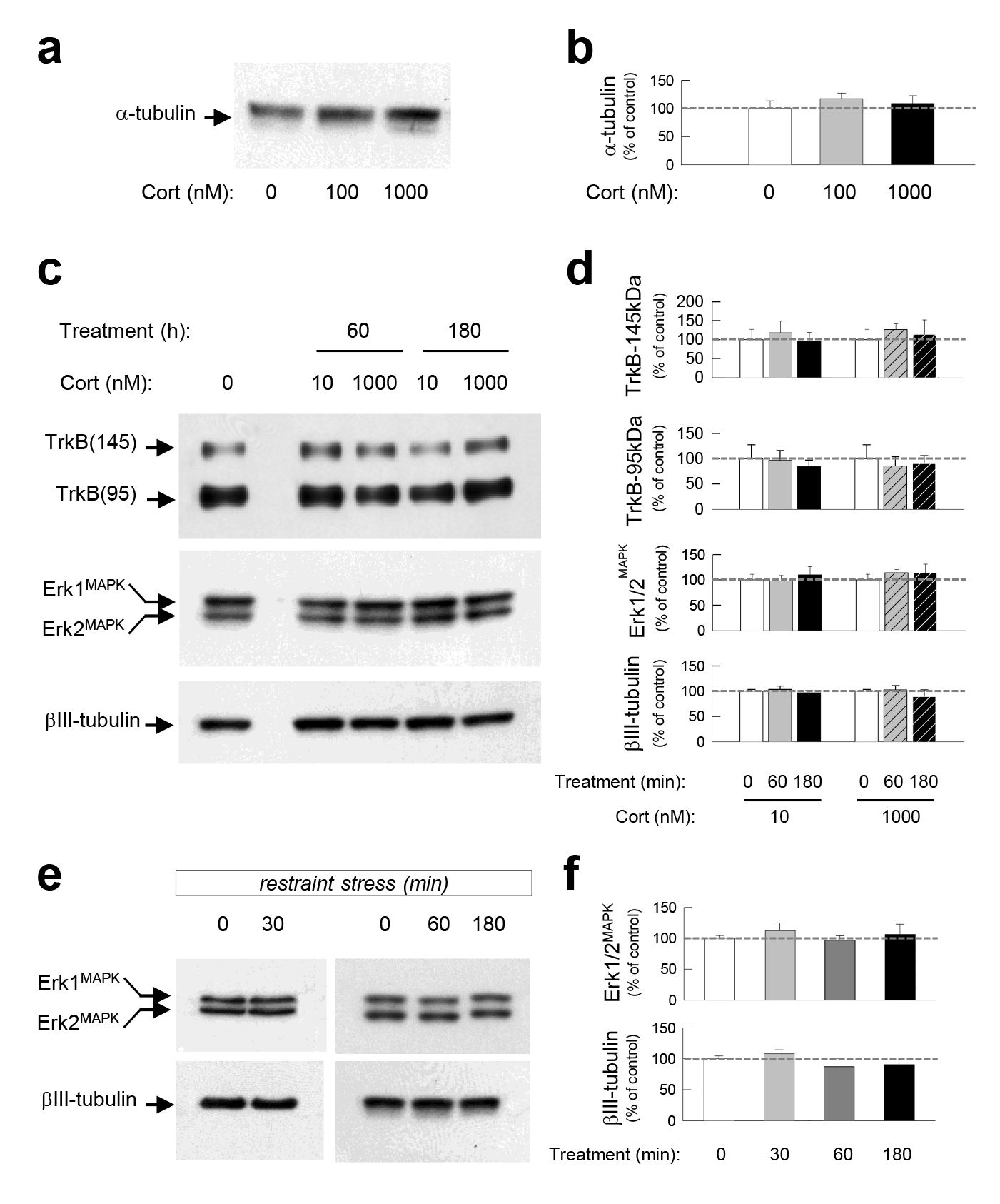

Supplement: Supplementary file 1 — Supp Figure S1 [file 41380_2021_1024_MOESM1_ESM.tif]

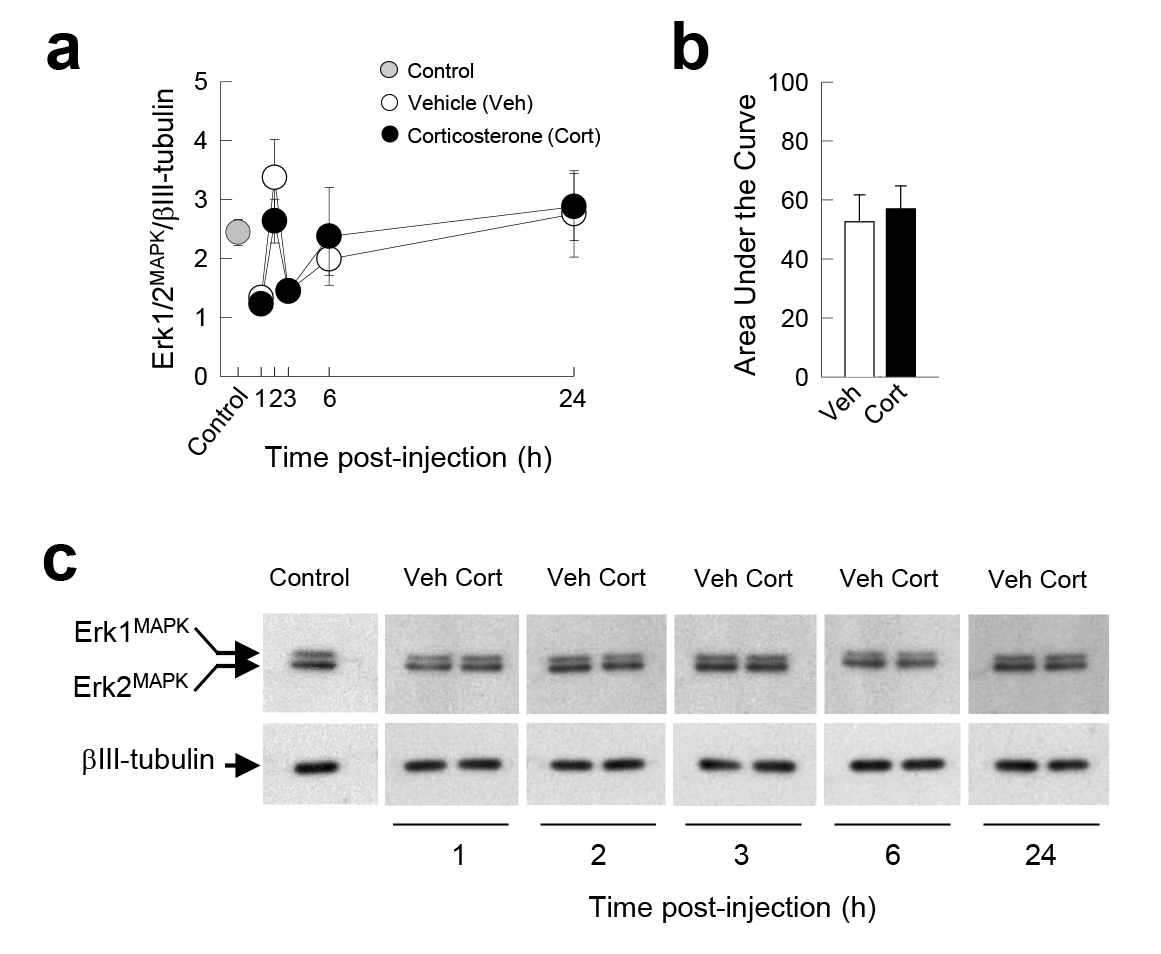

Supplement: Supplementary file 2 — Supp Figure S2 [file 41380_2021_1024_MOESM2_ESM.tif]
