# Supplementary material for: PAI-1 protein is a key molecular effector in the transition from normal to PTSD-like fear memory
Source: Mol Psychiatry. 2021 Jan 28;26(9):4968–81. doi: 10.1038/s41380-021-01024-1 (PMC8589667; doi:10.1038/s41380-021-01024-1)
Supplement: Supplementary file 3 — SUPPLEMENTARY INFORMATION [file 41380_2021_1024_MOESM3_ESM.pdf]

SUPPLEMENTARY INFORMATION

PAI-1 protein is a key molecular effector in the transition from normal to PTSD-like fear memory

C. Bouarab[1]†, V. Roullot-Lacarrière[1]†, M. Vallée[1], A. Le Roux[1], C. Guette[1], M. Mennesson[1], A. Marighetto[1], A. Desmedt[1]†, PV. Piazza[1,2]†, JM. Revest[1]†*.

[1] Univ. Bordeaux, INSERM, Neurocentre Magendie, U1215, F-33000 Bordeaux, France

[2] Current address: Aelis Farma, 33077 Bordeaux, France.

† These authors contributed equally to this work

* To whom correspondence should be addressed: E-mail: jean-michel.revest@inserm.fr

ORCID ID: 0000-0001-6459-0888

Supplementary Figure Legends

Fig. S1 Corticosterone and restraint stress did not modify the expression of total proteins. (**a**) Western Blot and (**b**) densitometric analysis of α-tubulin protein expression used as a loading control in PC12 cells in response to 100 nM and 1000 nM of Cort for 3 h (180 min). (**c**) Western Blot and (**d**) densitometric analyses of TrkB, Erk1/2^{MAPK} and βIII-tubulin proteins from dorsal hippocampal slices extracts of Sprague-Dawley rats incubated with 10 nM and 1000 nM of Cort for 1 h (60 min) and 3 h (180 min). (**e**) Western Blot and (**f**) densitometric analyses of Erk1/2^{MAPK} and βIII-tubulin protein expressions used as a loading control from dorsal hippocampus extracts from C57BL/6J mice in response to 30 min, 1 h (60 min) and 3 h (180 min) restraint stress. Dunnett's multiple comparisons *post-hoc* test after ANOVA, all *p*=ns. Plotted values are means +/- sem.

Fig. S2 The development of PTSD-like memory is not associated with changes in total proteins. Densitometric analyses of the expression of Erk1/2^{MAPK} protein in the dorsal hippocampus at different times after the conditioning sessions (**a**) and expressed as area under the curve encompassing the 24 h of analysis (**b**). Example of the Western Blot used for protein quantification after normalization with the level of βIII-tubulin (**c**). Immediately after the conditioning session animals received an injection of either vehicle (Veh; NaCl 0.9% i.p., white symbol) or of Cort (2 mg/kg i.p., black symbol). Grey symbol, control animals that were manipulated but not exposed to conditioning. Student’s t-test and Sidak's multiple comparisons *post-hoc* test after ANOVA, all *p*=ns. Plotted values are means +/- sem.
